# Histomorphological and ultrastructural analysis of vibrissal follicle-sinus complexes in the dog (*Canis lupus familiaris*)

**DOI:** 10.1371/journal.pone.0355554

**Published:** 2026-08-19

**Authors:** Helen Edith Müller, Sonja Fiedler, Eva Schwarz, Sophia Bliemel, Sophie Franzmeier, Andrea Fuchs-Baumgartinger, Balazs Gerics, Pauline Pöpperl, Rouven Wannemacher, Sven Reese, Dorothea Döring, Kaspar Matiasek, Andreas Blutke

**Affiliations:** 1 Institute of Veterinary Pathology, Center for Clinical Veterinary Medicine, Ludwig-Maximilians-Universität München, Munich, Germany; 2 Unit of Pathology, Department of Biological Sciences and Pathobiology, University of Veterinary Medicine Vienna, Vienna, Austria; 3 Unit of Morphology, Department of Biological Sciences and Pathobiology, University of Veterinary Medicine Vienna, Vienna, Austria; 4 Department of Pathology, University of Veterinary Medicine Hannover, Foundation, Hanover, Germany; 5 Chair of Anatomy, Histology and Embryology, Department of Veterinary Sciences, Ludwig-Maximilians-Universität München, Munich, Germany; 6 Chair of Animal Welfare, Ethology, Animal Hygiene and Husbandry, Ludwig-Maximilians-Universität München, Munich, Germany; Federal University of Rio de Janeiro, BRAZIL

## Abstract

Trimming of vibrissae in dogs (*Canis lupus familiaris*) for cosmetic reasons remains a common practice, particularly among poodle breeds and show dogs. In discussions surrounding this practice, it has been claimed that domestic dogs would have fewer and structurally less developed sinus hairs than other mammals, and that, especially in poodles, vibrissae would lack functional relevance, although such narratives represent popularized assumptions rather than systematically supported peer-reviewed evidence. The follicle-sinus complexes (FSCs) of various species, including cats (*Felis catus*), rats (*Rattus norvegicus*), and marine mammals, have been characterized in detail with respect to their innervation and mechanoreceptor organization. However, comparable histomorphological analyses of canine FSCs have not yet been conducted. Providing an in-depth morphological characterization of canine FSCs, the present study examined facial skin samples from 44 dogs of different ages, breeds (including poodles), and sexes, with assessment of FSC number and size. Regardless of breed, an average of 60 ± 13 FSCs per dog was identified in the present study (with a regional distribution of 28 ± 7 mystacial, 9 ± 2 supraorbital, 16 ± 7 mandibular, and 7 ± 2 buccal FSCs). Mystacial FSCs were the longest, measuring 4.0 ± 1.1 mm in length and 1.7 ± 0.6 mm in width. FSC length and width were significantly positively associated with body weight across all investigated facial regions. FSCs were characterized using histological, immunohistochemical (PGP 9.5, NF200, S100, GFAP), ultrastructural, and quantitative-morphological analyses. Canine FSCs demonstrate a complex mechanosensory architecture that includes Merkel cell-neurite complexes, lanceolate endings, reticular endings, and presumptive free nerve endings located in specific regions of the FSC. Quantitative analyses showed that neuronal tissue accounted for 13.6% ± 3.3% (vol.) of the internal FSC compartments. No evidence was found of a species- or breed-specific reduction in the structural or sensory capacity of canine FSCs.

## Introduction

Vibrissae are specialized tactile hairs present in the majority of mammalian species, including dogs [1]. Anchored in follicle-sinus complexes (FSCs), vibrissae function as specialized tactile organs that enable fine perception of environmental stimuli through dense mechanoreceptor innervation [2–8]. In dogs, vibrissae are arranged in specific patterns across different areas of the face, including the mystacial pad, buccal area, supraorbital ridge, and submental region [9,10].

For decades, it has been common practice to trim or shave dogs’ vibrissae for cosmetic reasons, especially in preparation for dog shows [11,12]. This practice is particularly prevalent in poodles, where muzzle shaving is commonly performed for (allegedly) aesthetic purposes. This is supported by an expert report proposing that canine vibrissae would be reduced in number, display less morphological differentiation, and have reduced sensory function in these breeds [13]. It has also been suggested that in breeds such as poodles, dense facial hair and selective breeding may have masked or further limited the functionality of vibrissae [13,14]. However, systematic evidence supporting these assumptions is lacking.

The structure and sensory architecture of FSCs have been extensively studied in species such as rats [2,3,5,15–17], cats [2,3,17], and marine mammals [18–20] using light microscopy, immunohistochemistry, and electron microscopy. Comparative studies have also examined the external vibrissal hair shaft and its movement in different species. In Carnivora, aquatic species have thicker, shorter, and smoother vibrissae, with fewer scales than terrestrial species [21]. Vibrissal movements have been documented in both aquatic carnivorans, such as the harbour seal (*Phoca vitulina*), and terrestrial carnivorans, including the red fox (*Vulpes vulpes*) [22], as well as in domestic dogs [27].

As described in several mammalian species, FSCs generally conform to a basic sinus-type organization, comprising the vibrissal hair follicle enclosed by a connective tissue capsule and associated with blood-filled sinus compartments, including the ring sinus and cavernous sinus [3]. Previous studies have demonstrated that the sensory innervation of mystacial FSCs is distributed across six distinct anatomical sites within the FSC, namely the rete ridge collar, i.e., the epidermal collar surrounding the follicular opening, the inner conical body, the outer root sheath, and the mesenchymal sheath at the level of the ring sinus and the cavernous sinus, as well as the dermal papilla [3]. However, comparable investigations in the domestic dog have not yet been conducted in greater detail. An early study by Ueda [23] examined canine vibrissae and follicles and reported a histological organization similar to that of cats. Subsequent studies have confirmed the presence of intrinsic musculature in mystacial vibrissae [24] and Merkel cells within the follicle [25,26]. A recent study demonstrated the behavioral relevance of vibrissae in dogs, highlighting their involvement in exploration, feeding, and protective responses [27]. Preliminary histomorphological and ultrastructural analyses of a limited number of dogs confirmed that canine FSCs exhibit the general architecture of mammalian sinusoidal vibrissae, including a dense innervation [27]. However, unlike in other species, detailed analyses of mechanoreceptor types and innervation density in canine FSCs, as well as the presence and organization of innervation across distinct anatomical sites of the follicle, have not yet been systematically investigated.

In the present study, we aimed to investigate whether the canine FSC is densely innervated and exhibits a complex mechanoreceptor architecture comparable to that described in other mammalian species in which vibrissae play a critical role in tactile perception, including tactile guidance of movements, avoidance behavior, and the expression of emotions.

## Materials and methods

### Tissue sample collection and ethics statement

**Fig 1:**
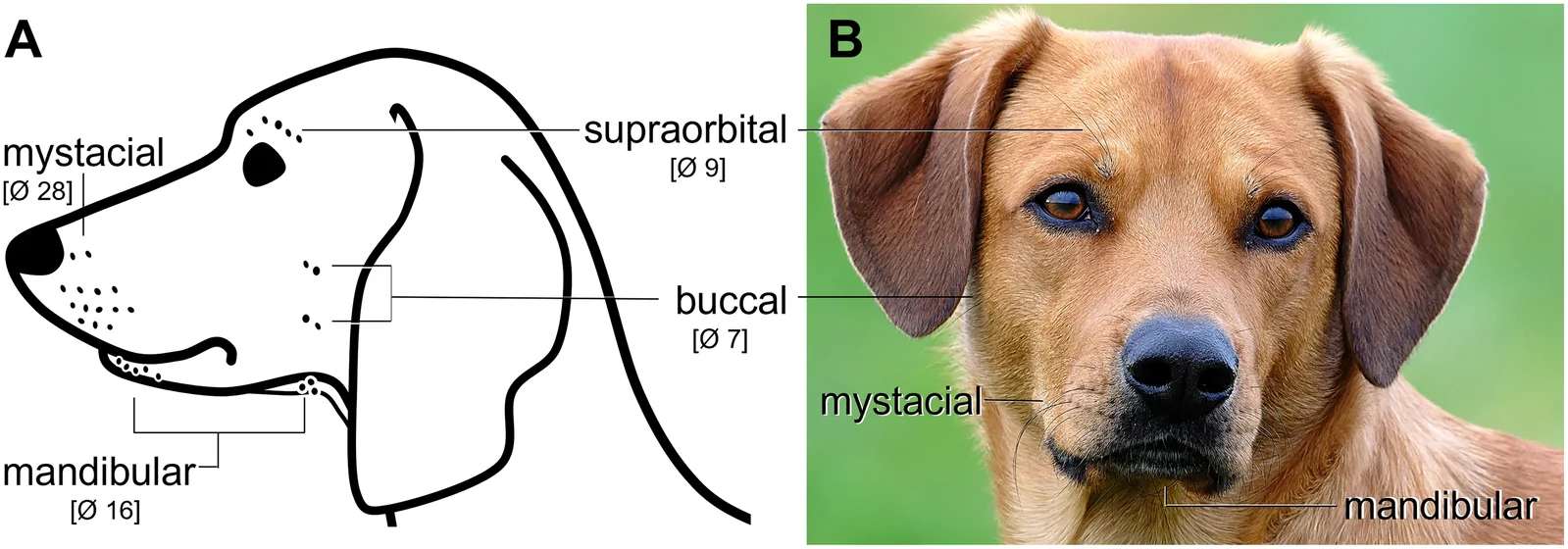
Location, classification used in the present study, arrangement, and mean numbers of identified canine facial (vibrissal) follicle-sinus complexes (FSCs). **(A)** Schematic illustration. **(B)** Photograph of an Austrian Pinscher, reproduced under a CC BY 4.0 license, with permission from C. Blechinger, original copyright 2024.

The use of animal tissues in this study was conducted in accordance with the German Animal Welfare Act and was approved by the institutional Ethics Committee of the Institute of Veterinary Pathology of the Ludwig-Maximilians-Universität München (LMU) (Protocol Number: EC1/26). Written informed owner consent was obtained for the use of submitted animal bodies and necropsy samples for research purposes. No animals were euthanized or sacrificed for the purpose of this study, and no procedures were performed on live animals as part of the present investigation. Facial skin samples were excised from fresh animal carcasses, i.e., from carcasses that had not been frozen or previously fixed, from 40 domestic dogs of various ages, breeds (mixed and purebred), and sexes during routine necropsies at the Institute of Veterinary Pathology, LMU. Additional tissue samples from four poodle and poodle-type dogs were obtained from the Department of Pathology, University of Veterinary Medicine Hannover, Foundation, Hannover, and from the Department of Biological Sciences and Pathobiology, University of Veterinary Medicine, Vienna, Austria. In total, samples from 44 dogs were included in the study, representing 31 distinct specific breed designations and 8 designations referring to mixed-breed or cross-breed dogs; sex was documented for 40 dogs, including 20 males and 20 females. A complete list of all examined cases and associated data, including breed or breed designation, age, sex, body weight, pathological diagnosis, and sampled facial region(s) is provided in the Supporting Information (S1 Table). Sample numbers varied across analyses depending on sample availability and suitability for the respective analysis, as specified below. Vibrissae were counted bilaterally at four facial locations (mystacial, supraorbital, buccal, and mandibular) (Fig 1), with buccal and zygomatic vibrissae grouped together as buccal, and lower labial and mental vibrissae grouped together as mandibular. The surrounding fur was first moistened to enable macroscopic identification from the outer aspect, followed by dissection of the facial skin to identify FSCs on the underside of the skin. Additionally, the morphology of the vibrissal hair shaft (curly or straight) was recorded. All dogs included in the study had untrimmed vibrissae. Only cases in which all four facial vibrissal regions were available were included in the analysis of total FSC number counts (n = 31). Tissue samples (approximately 5 mm × 5 mm × 15 mm) containing selected follicles (two FSCs per case and location) were excised [28] using a scalpel (No. 24), trimmed under a stereomicroscope (Carl Zeiss AG, Germany), and fixed for histological analyses. Follicle dimensions (maximal longitudinal extension and diameter, i.e., length and width) were measured in approximately sagittal midline sections of formalin-fixed, paraffin-embedded FSCs at four facial locations. For the analysis of mystacial FSCs, the most caudally located FSCs within the second or third mystacial row of the mystacial pad were excised, corresponding to the long caudal mystacial vibrissae described by Dougill et al. [21].

### Histomorphological analyses

Histomorphological evaluation by light microscopy was performed on all available FSC samples from each case, as listed in S1 Table (n = 44). The FSC samples were fixed in 4% neutral buffered formalin for more than 12 hours at room temperature with gentle agitation. To facilitate subsequent sectioning of fixed and paraffin-embedded tissue samples, FSCs were either treated with a commercially available depilatory cream (ISANA^®^, Dirk Rossmann GmbH, Germany) for 90 minutes, followed by mechanical removal of any residual cream by washing in Tris-buffered saline (TBS) and careful manual extraction of the hair shafts using fine tweezers, or by incubation for 48 hours in a Titriplex-based solution (100 g Titriplex^®^ III (Merck KGaA, Germany) and 33 g TRIS (Sigma 7–9^®^, Merck KGaA, Germany) dissolved in distilled water). To stabilize the samples mechanically during the subsequent paraffin-embedding procedure and for section orientation, the FSC samples were embedded in warm, polymerizing 2% agar (Kobe I, Karl Roth GmbH & Co. KG, Germany) [29]. After paraffin embedding, sections were cut at nominal thicknesses of 2.0 µm or 2.5 µm using a fully automated rotary microtome (Epredia™ HM 355S with Section Transfer System™ (STS), Thermo Fisher Scientific, Germany), mounted on StarFrost^®^ glass slides (Engelbrecht GmbH, Germany), and stained with hematoxylin and eosin (HE) following standard protocols.

A detailed step-by-step illustration of the histotechnical processing of FSC samples is provided in the Supporting Information (S1 Fig).

### Immunohistochemical analyses

Immunohistochemical detection of neural marker proteins within the follicle-sinus complex (FSC) was performed on formalin-fixed, paraffin-embedded tissue sections obtained from four facial regions (mystacial, supraorbital, buccal, and mandibular) using specific primary and secondary antibodies (Table 1).

**Table 1:** Primary and secondary antibodies used for immunohistochemical detection of neural markers in canine formalin-fixed, paraffin-embedded FSC tissue samples.

Target protein	Antibody	Marker properties
**PGP 9.5** protein gene product 9.5	Polyclonal rabbit anti-PGP 9.5 primary antibody (Code No. Z5446, Dako Cytomation, Denmark; dilution 1:100)	Anti-PGP 9.5 has been established as a pan-axonal/neuronal marker in the FSC across various species, including rodents [15,17,30,31], cats [17], Florida manatees (*Trichechus manatus latirostris*) [20], rock hyraxes (*Procavia capensis*) [28], and dogs [27]. PGP 9.5 has also been shown to detect Merkel cells in rats [16,17], cats [17], and rock hyraxes [28].
**S100** S100 protein	Polyclonal rabbit anti-S100 primary antibody (Code No. Z0311, Dako Cytomation, Denmark; dilution 1:600)	Anti-S100 has been used as a glial marker in the FSCs of rats [15,30], Florida manatees [20], and rock hyraxes [28]. In dogs, S100 immunoreactivity was also observed in the cytoplasm of Merkel cells [25].
**NF200** Neurofilament 200	Monoclonal mouse anti-NF200 primary antibody (Code No. N0142, Sigma-Aldrich, USA, dilution 1:1000)	Anti-NF200 immunolabeling of myelinated nerve fibers has been reported within the FSC of various species, including rats [15], Florida manatees [20], and rock hyraxes [28].
**GFAP** Glial fibrillary acidic protein	Polyclonal rabbit anti-GFAP primary antibody (Code No. Z0334, Dako Cytomation, Denmark, dilution 1:600)	Anti-GFAP immunostaining was used to identify GFAP-expressing peripheral glial cells, including non-myelinating Schwann cells, as previously described in rodents [32–35], and has also been reported to label dermal nerve processes in dogs [25].
Goat anti-rabbit IgG antibody	Biotinylated goat anti-rabbit IgG antibody (BA-1000, Vector, Peterborough, UK, dilution 1:200)	
Horse anti-Mouse IgG antibody	ImmPRESS^®^ HRP Horse Anti-Mouse IgG Polymer Reagent (MP-7402, Vector Laboratories, USA)	

Immunohistochemical detection of protein gene product 9.5 (PGP 9.5), S100 protein (S100), and glial fibrillary acidic protein (GFAP) was performed using the avidin-biotin-peroxidase method. Neurofilament 200 (NF200) was detected using a polymer-based peroxidase system (ImmPRESS^®^ HRP Horse Anti-Mouse IgG Polymer Detection Kit, MP-7402, Vector Laboratories, USA). The sections were deparaffinized in 100% xylene, rehydrated through a graded ethanol series, and rinsed in distilled water. Endogenous peroxidase activity was blocked using 3% (w/v) hydrogen peroxide (10 vol.), prepared by tenfold dilution of 30% (w/v) hydrogen peroxide, 100 vol. (AppliChem, Germany), for 15 minutes. For NF200, blocking was performed using 3% hydrogen peroxide in methanol for 30 minutes. Non-specific binding was prevented using a serum-based blocking solution (normal goat serum (MP Biomedicals, USA) 2.5% supplemented with avidin (Avidin/Biotin Blocking Kit, VEC-SP-200, Vector Laboratories, USA)) or 2.5% normal horse serum (S-2012, Vector Laboratories, USA) for the detection of NF200 for 30 minutes. Sections were incubated with the respective primary antibodies at the dilutions specified in Table 1 in Tris-buffered saline (TBS), with biotin supplementation for PGP 9.5, S100, and GFAP (Avidin/Biotin Blocking Kit, VEC-SP-200, Vector Laboratories, USA). The slides were then incubated with primary antibodies for either 12 hours at 4 °C (S100, GFAP, NF200) or for 60 minutes at room temperature (PGP 9.5). After rinsing three times in TBS, the sections were incubated with the appropriate secondary antibody diluted in TBS for 50 minutes. For PGP 9.5, S100, and GFAP, this was followed by an incubation with an avidin/biotin-based peroxidase system (Vectastain^®^ Elite ABC-Kit, Peroxidase (HRP), Cat. No. PK-6100, Vector Laboratories, USA) for 30 minutes. Immunoreactivity was visualized using 3,3′-diaminobenzidine tetrahydrochloride (ImmPACT^®^ DAB Substrate, Peroxidase, SK-4105, Vector Laboratories, USA) as chromogen. The sections were then counterstained with hematoxylin, dehydrated through an ascending ethanol series, cleared in xylene, and cover-slipped with Histokitt^®^ (Engelbrecht, Germany). Staining specificity was confirmed using formalin-fixed, paraffin-embedded canine spinal ganglia tissue as a positive control. Negative control slides were incubated with TBS instead of the primary antibody.

### Quantitative-morphological analysis of nerve tissue density in canine FSCs

Paraffin-embedded longitudinal sections through the center of mystacial FSCs immunolabeled for PGP 9.5 were used for quantitative analysis of the area density of PGP 9.5-positive nerve tissue in the FSC, using the point-counting method [36,37] as previously described [38–42]. One PGP 9.5-immunolabeled longitudinal section per case was analyzed in 24 cases that fulfilled the predefined section-plane criteria, namely the presence of a complete central longitudinal profile of a mystacial FSC. The fractional section profile areas of PGP 9.5-positive fibers were determined within five distinct, anatomically defined FSC tissue compartments: the mesenchymal sheath (MS), glassy membrane (GM), trabeculae (Trab), ringwulst (RW), and inner conical body (ICB), as the ratio of grid points hitting PGP 9.5-positive nerve fiber profiles to the total number of grid points hitting the FSC tissue. Analyses were performed using an automated stereology system (VIS-Visiopharm Integrator System^TM^, Version 2021.09, Visiopharm A/S, Denmark) at 40 × magnification, with a 100% sampling fraction, utilizing superimposed 16 × 16 point grids. On average, 3803 ± 1877 points were counted in 58 ± 21 sampled fields of view per FSC.

### Ultrastructural analyses

For transmission electron microscopy (TEM) analyses, FSC samples of approximately 1 mm³ were excised from follicles of four facial vibrissal regions (mystacial, buccal, supraorbital, and mandibular) and fixed in 2.5% glutaraldehyde in 0.1 M Sørensen’s phosphate buffer solution (pH 7.4). The samples were then post-fixed in 1% osmium tetroxide buffered according to Caulfield [43]. Dehydration was performed using a graded acetone series, followed by infiltration and embedding in glycidyl ether-based epoxy resin (prepared from Glycid ether 100 (Epon 812) with 2-Dodecenylsuccinic acid anhydride (DDSA), methyl nadic anhydride (MNA), and 2,4,6-tris(dimethylaminomethyl)phenol (DMP-30), SERVA Electrophoresis GmbH, Germany) [44]. Polymerization was carried out in flat embedding molds at 60 °C for 48 h. The polymerized blocks were trimmed (Ultratrim TM60, Reichert Jung, Austria), and semithin sections with a nominal thickness of 0.5 μm were cut with an ultracut rotary microtome (Ultracut E, Reichert Jung, Austria) equipped with a histo-/ultra-diamond knife (Diatome Ltd, Switzerland). The sections were mounted on glass slides (ISO 8037−1, R. Langenbrinck GmbH, Germany), and routinely stained with toluidine blue (Karl Roth GmbH & Co. KG, Germany) and Safranin O (Waldeck GmbH & Co. KG, Germany) to visualize nerves in epoxy resin-embedded tissue [45]. For TEM analysis, ultrathin sections of 70 nm nominal thickness were cut with an ultracut rotary microtome (Ultracut E, Reichert Jung, Austria), mounted on copper grids (Veco B.V., Netherlands), stained with uranyl acetate and lead citrate according to Reynolds [46], and examined at different magnifications using a transmission electron microscope (EM 109/900, Carl Zeiss AG, Germany) [40].

### Statistical analysis

Quantitative data are presented as mean ± SD. Quantitative variables included FSC length, FSC width, mandibular and total FSC counts, PGP 9.5-positive nerve tissue area density, body weight, and age. Categorical grouping variables included sex, head morphotype, and breed group, as indicated in S1 Table. Normality of quantitative variables was assessed using the Shapiro-Wilk test. For group comparisons, homogeneity of variances was assessed using the F test for two-group comparisons and the Brown-Forsythe test for comparisons among more than two groups. Associations between quantitative variables were analyzed using Spearman’s rank correlation. Differences between two independent groups were analyzed using the Mann-Whitney *U* test, whereas comparisons among more than two independent groups were performed using the Kruskal-Wallis test. Statistical analyses were performed using GraphPad Prism version 10.6.1 for Windows (GraphPad Software, www.graphpad.com). *p* values < 0.05 were considered statistically significant.

*P* values, sample sizes, statistical tests, and test statistics are provided in S2 Table.

## Results

### Number, distribution, and size of canine follicle-sinus complexes (FSCs)

The number of identified FSCs varied among individuals while maintaining region-specific bilateral patterns in the mystacial (28 ± 7), supraorbital (9 ± 2), mandibular (16 ± 7), and buccal (7 ± 2) regions in the 31 cases in which all four facial vibrissal regions were completely available for analysis (Fig 1). Among these regions, mandibular FSC counts showed the greatest relative interindividual variability (coefficient of variation approximately 46%). Mandibular FSC counts were not significantly correlated with age, body weight, or mandibular FSC length (*r* = 0.0493–0.1365, all *p* > 0.05; S2 Table) and did not differ significantly according to sex, head morphotype, or breed group (all *p* > 0.05; S2 Table). Total FSC counts were likewise not significantly correlated with age or body weight (*r* = 0.1215–0.1864, both *p* > 0.05; S2 Table) and did not differ significantly according to sex, head morphotype, or breed group (all *p* > 0.05; S2 Table). The mystacial FSCs displayed the greatest length (4.0 ± 1.1 mm in length; 1.7 ± 0.6 mm in width, *n* = 43), followed by the buccal (3.8 ± 1.0 mm × 1.8 ± 0.6 mm, n = 39), mandibular (3.8 ± 1.0 mm × 1.7 ± 0.5 mm, *n* = 34), and supraorbital FSCs (3.1 ± 0.8 mm × 1.4 ± 0.4 mm, *n* = 41). In cases with available body weight data, FSC length and width were significantly positively associated with body weight across all investigated facial regions (all *p* < 0.001; S2 Table). The vibrissal hair shaft morphology varied, with curly vibrissae predominantly observed in dogs with curly or wavy coats, whereas dogs with straight, long, or wire-haired coats generally exhibited straight vibrissal hair shafts (S1 Table, S2 Fig).

### Histomorphology of canine FSCs

**Fig 2:**
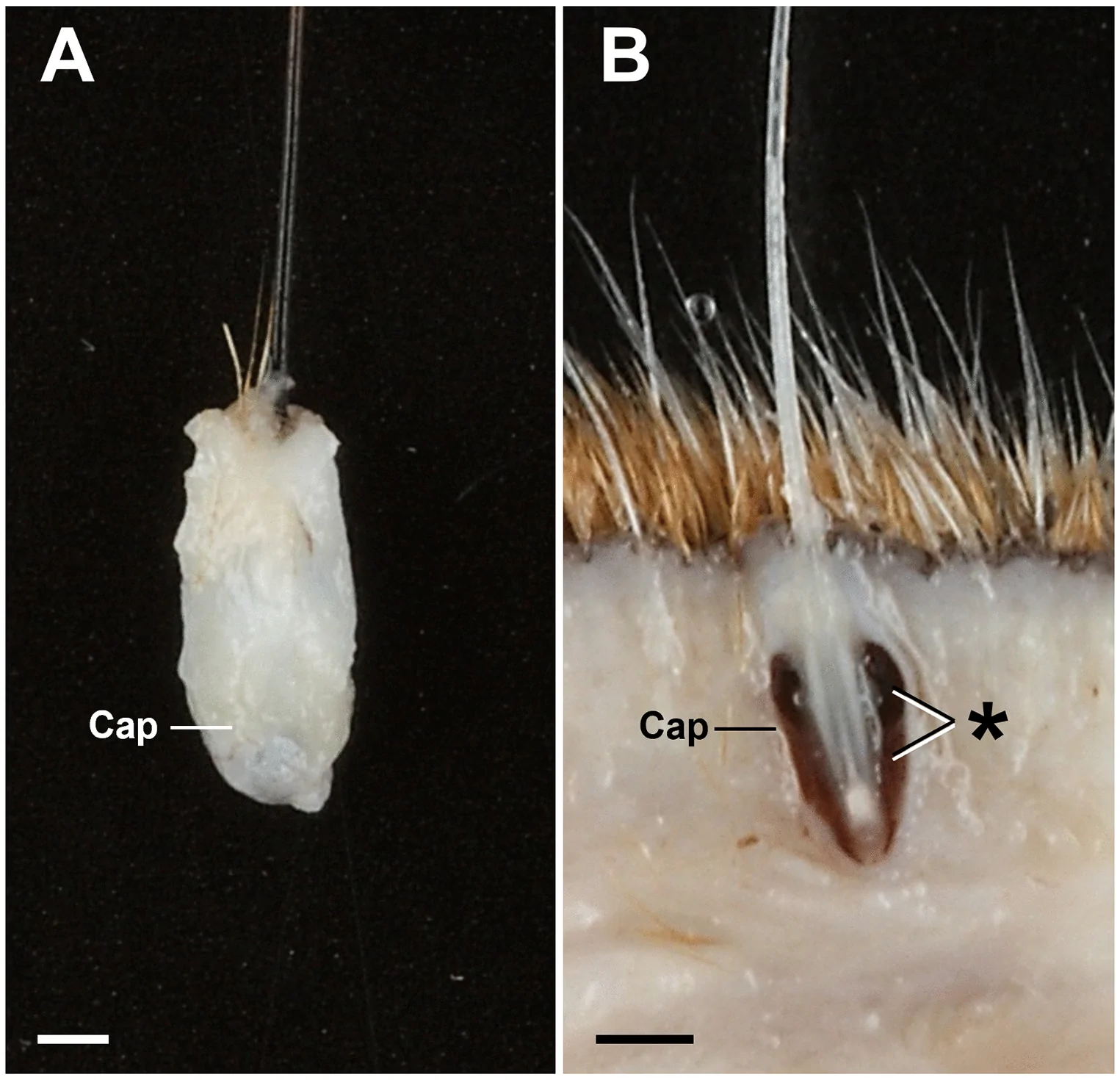
Gross morphology of canine mystacial follicle-sinus complexes (FSCs). (**A)** Excised FSC surrounded by a thick connective tissue capsule (Cap). (**B**) Sagittal midline FSC-section with blood-filled sinus (*) surrounding the vibrissal hair follicle. Bars = 1 mm.

**Fig 3:**
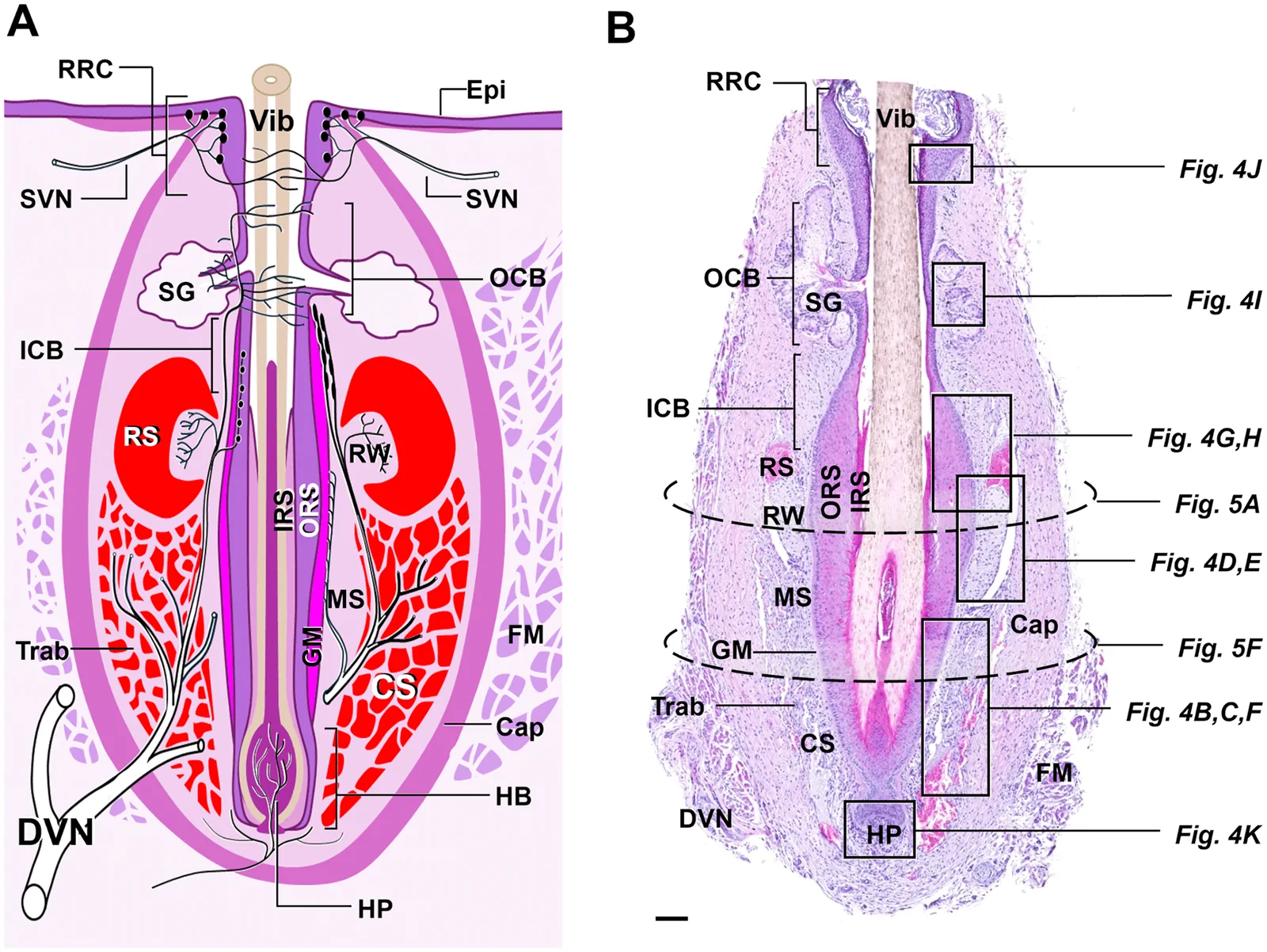
Morphology and innervation of the canine (vibrissal) follicle-sinus complex (FSC). **(A)** Schematic illustration. **(B)** Mystacial FSC histology (Rhodesian Ridgeback, male, 5 weeks of age; No. 37, S1 Table). The hair shaft **(Vib)** is surrounded by an internal root sheath (**IRS**) and enveloped by the epithelium of the outer root sheath (**ORS**). The rete ridge collar (**RRC**) surrounds the hair shaft as it exits the follicle. Below the rete ridge collar (**RRC**), the follicle is surrounded by the outer (**OCB**) and the inner conical body (**ICB**). Multilobulated sebaceous glands (**SG**) encircle the follicle at the level of the outer conical body (**OCB**). The blood sinus is subdivided into the ring sinus (**RS**), characterized by a prominent “ringwulst” (**RW**) protruding into its lumen, and the cavernous sinus (**CS**), traversed by connective tissue trabeculae (**Trab**). The “glassy membrane” (**GM**) separates the outer root sheath (**ORS**) from the mesenchymal sheath (**MS**) and is thickened at the level of the ring sinus (**RS**), remaining prominent throughout the cavernous sinus (**CS**). A dense collagenous capsule (**Cap**) encloses the **FSC**. In mystacial FSCs, the follicular (intrinsic) muscle (**FM**) attaches to the follicle capsule from the level of the ring sinus (**RS**) to the lower third of the cavernous sinus (**CS**). Thin nerve bundles penetrate the base of the **FSC** to innervate the hair papilla (**HP**). The deep vibrissal nerve (**DVN**) bifurcates before penetrating the follicle capsule, sending fibers into the trabeculae (**Trab**) and ascending within the mesenchymal sheath (**MS**), from which fibers project as nerve endings terminating along the outer surface of the glassy membrane (**GM**). At the upper level of the ring sinus (**RS**), nerve fibers cross the glassy membrane (**GM**) to innervate Merkel cell-neurite complexes within the outer root sheath (**ORS**). Fine nerve fibers within the ringwulst (**RW**) become increasingly dense toward its margin facing the ring sinus (**RS**). Large, longitudinally oriented fibers terminate adjacent to the glassy membrane (**GM**) at the lower extent of the inner conical body (**ICB**) and the upper extent of the ring sinus (**RS**). A dense circumferential array of small-caliber fibers innervates the inner conical body (**ICB**). Within the outer conical body (**OCB**), thin nerve fibers encircle the sebaceous glands (**SG**) and their ducts. At the level of the rete ridge collar (**RRC**), Merkel cells are located in the basal layer of the epidermis (**Epi**), and a diffuse network of small-caliber fibers extends into the connective tissue, both of which are supplied by superficial vibrissal nerves (**SVN**). Boxed areas indicate regions further characterized in Figs 4 and 5. HE-stained paraffin section of formalin-fixed tissue. Bar = 100 µm.

**Fig 4:**
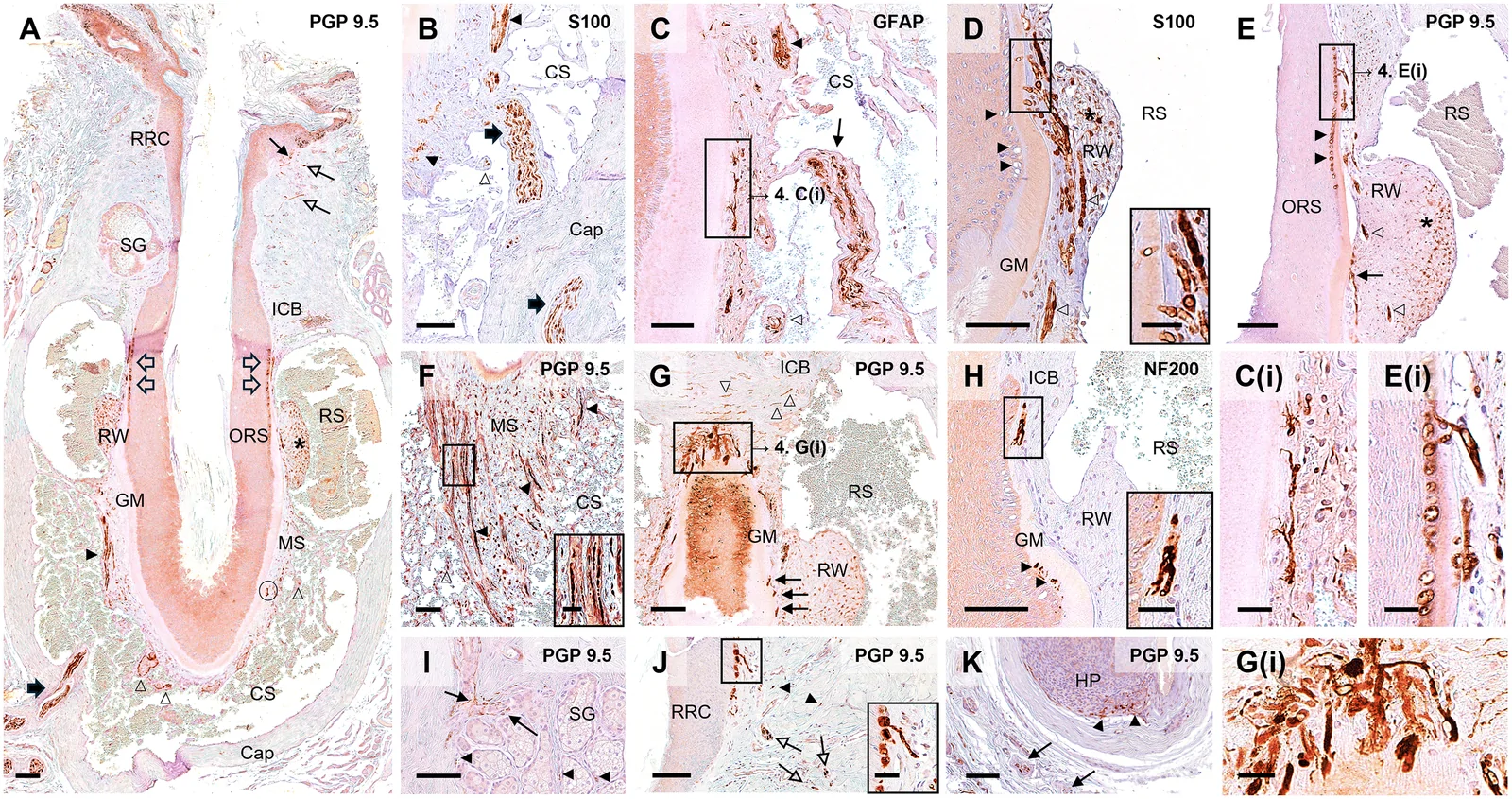
Innervation of the canine follicle-sinus complex (FSC). (**A)** Overview of the FSC (mandibular FSC, Rough Collie; No. 14, S1 Table) labeled with **PGP 9.5** (pan-neuronal marker), showing the main structural regions and the distribution of innervation within the follicle. Corresponding details are shown in **(B–K). (B), (C), and (F)** Cavernous sinus (CS) detail views. (**B) S100** (glial marker) (buccal FSC, Poodle cross-breed; No. 7, S1 Table). (**C) GFAP** (marker for GFAP-expressing peripheral glial cells) (supraorbital FSC, Berger Blanc Suisse; No. 17, S1 Table). (**F) PGP 9.5** (mandibular FSC, Retriever cross-breed; No. 11, S1 Table). The deep vibrissal nerve penetrates the capsule (Cap, broad solid arrow in A and B) in the lower third of the CS and attaches to the mesenchymal sheath (MS), ensheathed by connective tissue (thin arrow in C). Fibers extend into the trabeculae (open arrowheads, also in A) and the MS (solid arrowheads, also in A), where they ascend in a beaded, pearl-like pattern (inset in F) and form highly branched endings that terminate directly at the glassy membrane (GM; C(i), circle in A). **(D) and (E)** Ringwulst (RW) detail views. (**D) S100** (mystacial FSC, Poodle cross-breed; No. 7, S1 Table). (**E) PGP 9.5** (mystacial FSC, German Shepherd; No. 15, S1 Table). Large-caliber fibers ascend toward and within the RW (open arrowheads), cross the GM (inset in D and E(i)), and terminate as Merkel cell-neurite complexes in the outer root sheath (ORS, E(i), broad open arrows in A), where prominent vacuole-like spaces are present (solid arrowheads). Fine nerve fibers, **PGP 9.5**- and **S100**-positive, are present within the RW, with increasing density toward its outer margin facing the ring sinus (RS, asterisk, also in A). **(G) and (H)** Inner conical body (ICB) and upper RW detail views. **(G) PGP 9.5** (supraorbital FSC, Great Dane; No. 42, S1 Table). **(H) NF200** (marker for myelinated axons) (buccal FSC, Havanese; No. 25, S1 Table). In the ICB, fine-caliber fibers form a circumferential network (open arrowheads in G), while larger, longitudinally oriented fibers align in a palisade-like pattern along the GM (G(i) and inset in H). **NF200**-positive nerve fibers are also detected at the GM-ORS junction (solid arrowheads in H), likely contributing to the afferent innervation of Merkel cell-neurite complexes in the ORS. Where the RW attaches to the GM, medium-caliber **PGP 9.5-**positive fibers project toward the GM from the RW side (arrows in G, also in E). **(I)** Sebaceous gland (SG) detail view, **PGP 9.5** (buccal FSC, Magyar Vizsla; No. 31, S1 Table). Fine-caliber fibers surround the SG (arrowheads), and thin fibers encircle its ducts (arrows). **(J)** Rete ridge collar (RRC) detail view, **PGP 9.5** (mystacial FSC, Saint Bernard; No. 40, S1 Table). PGP 9.5-positive Merkel cells located in the basal epidermal layer at the RRC (inset, solid thin arrow in A) are supplied by superficial vibrissal nerves (open thin arrows, also in A), with fine-caliber fibers present in the surrounding connective tissue (arrowheads). **(K)** Hair papilla (HP) detail view, **PGP 9.5** (mandibular FSC, English Pointer cross-breed; No. 36, S1 Table). Numerous fiber bundles penetrate the base of the FSC (arrows), forming a meshwork of fine-caliber fibers innervating the HP (arrowheads). Sagittal midline sections immunolabeled with different primary antibodies (as indicated). DAB chromogen (brown); hematoxylin counterstain. Bars = 100 µm; inset bars = 25 µm.

**Fig 5:**
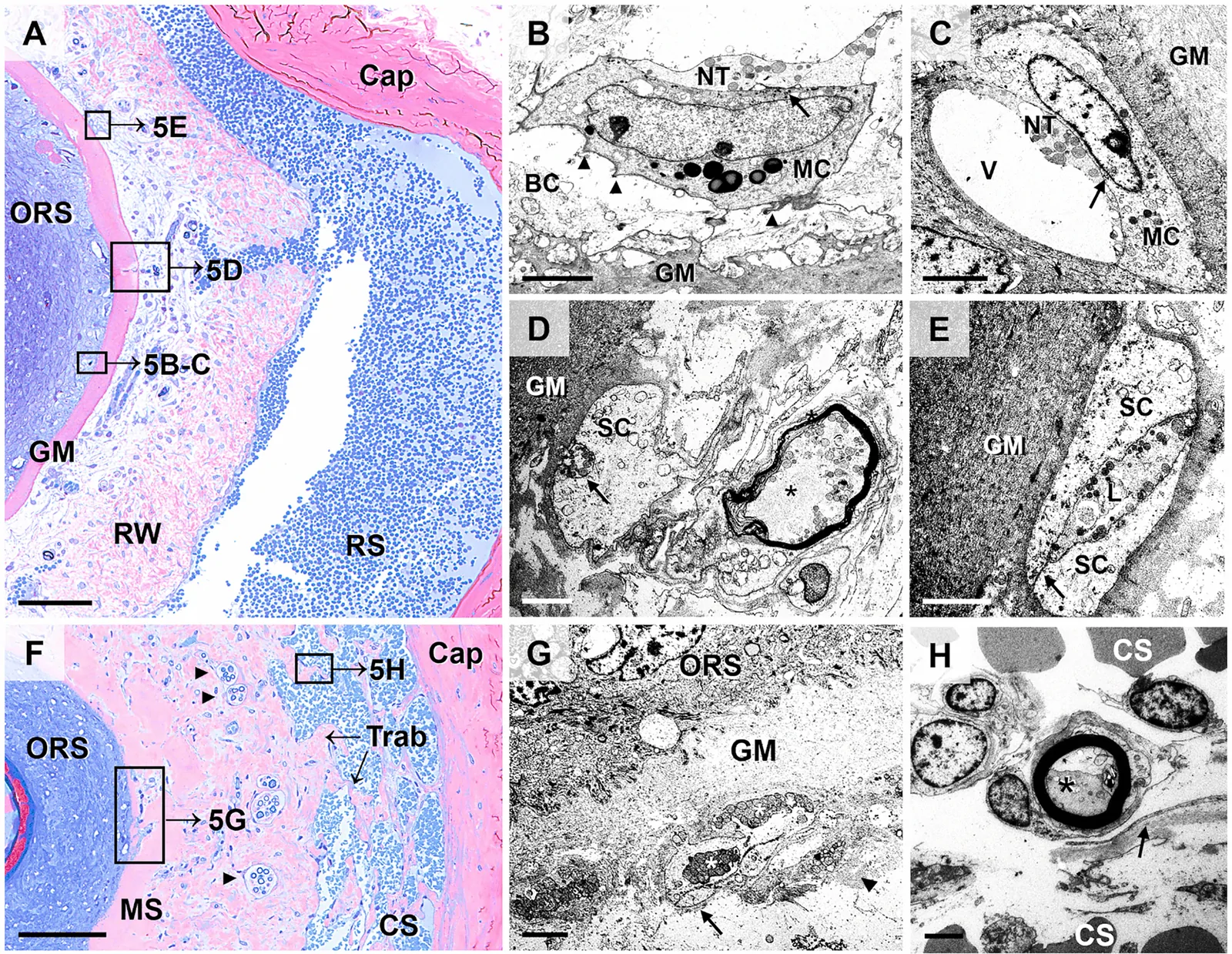
Ultrastructure of the canine follicle-sinus complex (FSC). **(A)** Mystacial FSC cross-section (Magyar Vizsla; No. 31, S1 Table) at the level of the ring sinus (RS) showing the ringwulst (RW) with a markedly thickened glassy membrane (GM) at this level, the outer root sheath (ORS), and the surrounding capsule (Cap). **(B, C) Merkel cells** (MC) in the canine FSC. MCs are located between cytoplasmic processes of basal cells (BC) of the ORS, near the GM, and are contacted by a nerve terminal (NT) on the side opposing the GM. They exhibit numerous dense-core neurosecretory granules (arrows) and elongated, finger-like cytoplasmic extensions (arrowheads) projecting toward BCs. **(C) Vacuole-like spaces** (V) are observed in direct contact with the NT, a feature previously reported only in aquatic mammals. **(D)** A **myelinated nerve** (asterisk) consisting of Schwann cell (SC)-ensheathed fibers (arrow) that project toward the GM. **(E)** An elongated, flattened **lanceolate ending** (L) runs parallel to the GM, giving it a characteristic sharp-edged appearance between Schwann cells (SC) and the surrounding connective tissue. The SC sheath is discontinuous at the pointed end facing the GM (arrow). **(F)** Buccal FSC cross-section (Poodle cross-breed; No. 7, S1 Table) at the level of the cavernous sinus (CS) showing a prominent mesenchymal sheath (MS) containing numerous ascending myelinated nerve fiber bundles (arrows). Trabeculae (Trab) are anchored to the MS. Numerous nerve fibers exhibit complex branching patterns as they approach the glassy membrane (GM), consistent with **reticular endings**. **(G)** On an ultrastructural level, these endings appear as **terminal axons** (asterisks) that are partially ensheathed by cytoplasmic lamellae of Schwann cells (arrow) and externally surrounded by collagen fibers (arrowhead). **(H)** Myelinated nerve fiber (asterisk), situated within a trabecula formed by fibroblast processes (arrow) traversing the CS. Toluidine blue and Safranin O-stained semithin sections of epoxy resin-embedded tissue (A, F; bars = 100 µm) provide orientation for the corresponding transmission electron micrographs shown in (B-E, G-H; bars = 2.5 µm).

In all histologically examined dogs (n = 44; S1 Table), and irrespective of breed, age, sex, and facial region, canine FSCs generally conformed to the basic sinus-type organization (Figs 2, 3, and S3 Fig), as described in other mammals [3]. No major breed-, sex-, age-, body weight-, or region-associated qualitative deviations from this basic FSC architecture were observed. The vibrissal hair follicle is surrounded by a thick connective tissue capsule (Cap), with maximum thickness at the level of the cavernous sinus (CS) (up to 500 µm). Striated muscle fibers insert laterally into the middle and lower thirds of the follicle capsule in FSCs of all examined facial regions. Within the capsule, two connected, blood-filled sinuses surround the vibrissal follicle: a distal cavernous sinus (CS), which is traversed by fine connective tissue trabeculae (Trab) originating from the (outer) follicle capsule and the (inner) mesenchymal follicle sheath (MS), and a proximal circular ring sinus (RS), at the mid-height of the follicle. At its medial aspect, the ring sinus envelops a mesenchymal bulge around the follicle, called the “ringwulst” (RW). The upper part of the follicle, where the vibrissal hair shaft exits the follicle opening, is surrounded by the rete ridge collar (RRC), distally followed by the outer (OCB) and inner conical body (ICB). At the level of the OCB, the follicle is completely encircled by multilobulated sebaceous glands (SG), whose ducts open at this level into the interval between the follicle and the vibrissal hair shaft. The follicular epithelium that envelops the vibrissal hair shaft consists of an inner and outer root sheath. A homogeneous, eosinophilic, hyaline basement membrane, the “glassy membrane” (GM), separates the outer root sheath epithelium of the hair follicle from the surrounding dermal connective tissue of the inner conical body, the ringwulst, and the mesenchymal follicle sheath. At the level of the ring sinus, this glassy membrane exhibits a notable thickening of up to 70 µm (Figs 4D and 5A). Finally, the distal end of the follicle is formed by the hair papilla (HP).

### Nerve supply to the canine FSC

The basic innervation pattern of the FSC is provided by three histomorphologically distinguishable nerve pathways, as illustrated schematically in Fig 3A.

Each FSC is innervated by a single, thick, **deep vibrissal nerve (DVN)** that regularly bifurcates before bilaterally entering the follicle’s connective tissue capsule, approximately at the level of the lower third of the cavernous sinus (Figs 3A, 4B, and S4 Fig). In the dermal connective tissue surrounding the follicle epithelium, the DVN branches further, forming intratrabecular nerve fibers in the cavernous sinus, and a network of fibers in the ringwulst (Figs 3A and 4B-E). At the level of the cavernous and the ring sinuses, additional DVN branches project to the interface of the mesenchymal sheath and the glassy membrane (Figs 3A and 4C-G), and extend to the level of the inner conical body and the excretory ducts of the sebaceous glands at the level of the outer conical body (Figs 3A, 4G, and 4I).

The innervation of the rete ridge collar (i.e., the uppermost part of the hair follicle infundibulum, where the hair shaft exits the follicle) is provided by **superficial vibrissal nerves (SVNs)** (Figs 3A and 4J).

Finally, the **hair papilla** is innervated by additional, fine-caliber nerve fiber bundles that enter the FSC at its base (Figs 3A and 4K).

### Innervation patterns, immunohistochemical and ultrastructural characterization of FSC nerve fibers and mechanoreceptors in defined follicle regions

#### Innervation of the rete ridge collar (RRC) and the outer conical body (OCB).

Within the **rete ridge collar (RRC)**, large-caliber, myelinated PGP 9.5- and NF200-positive nerve fibers extend toward the epidermis of the outer root sheath (ORS), where PGP 9.5-positive **Merkel cells** are present (Figs 3A and 4J). These cells are characterized by their typical localization within epidermal derivatives of the ORS and by their morphology, appearing as round to oval cells with cytoplasmic PGP 9.5 immunoreactivity, consistent with previous descriptions of vibrissal FSCs [3,17,28]. Additional (PGP 9.5- and GFAP-positive, NF200-negative) fine-caliber fibers extend into the subepithelial connective tissue of the RRC (Figs 3A and 4J).

Within the **outer conical body (OCB)**, fine-caliber nerve fibers, identified by PGP 9.5 immunoreactivity, absence of NF200-labeling, and occasional association with GFAP- and S100-positive glial elements, surround the multilobulated sebaceous glands and their excretory ducts (Figs 3A and 4I).

### Innervation of the inner conical body (ICB) and the middle portion of the follicle at the level of the ring sinus (RS)

A dense, circumferential network of fine-caliber nerve fibers is observed within the **inner conical body (ICB)**. These fibers are immunoreactive for PGP 9.5 and GFAP but lack NF200 immunoreactivity, consistent with free nerve endings (FNEs) (Figs 3A and 4G). No transversely oriented lanceolate endings are present in the examined FSCs.

Additionally, large longitudinal nerve endings positive for PGP 9.5, S100, GFAP, and NF200 form a palisade-like arrangement within the mesenchymal sheath (MS), adjacent to the glassy membrane (GM), at the level of the lower ICB and upper RS (Figs 3A, 4G, and 4H). TEM reveals that these nerve endings have the ultrastructural morphology of sharp-edged **lanceolate endings** interposed between Schwann cell processes (Fig 5E).

Notably, large-caliber nerve fibers positive for PGP 9.5-, NF200-, GFAP-, and S100 originate from the deep vibrissal nerve (DVN) and ascend within the ringwulst (RW). At the level of the upper **RS**, these fibers traverse the glassy membrane (GM) and project into the outer root sheath (ORS), where they innervate **Merkel cell-neurite complexes** (Figs 3A, 4D, 4E and 4H), identified by their characteristic localization between basal cells of the outer root sheath and the interface with the glassy membrane, as well as PGP 9.5 immunoreactivity and their characteristic ultrastructural morphology (Figs 5B and 5C). Interestingly, prominent vacuole-like spaces are frequently observed within the outer root sheath directly adjacent to the glassy membrane at the level of the lower ICB and upper RS and are found adjacent to PGP 9.5-immunoreactive Merkel cell-neurite complexes (Figs 4D and 4E). Ultrastructural analysis revealed oval to round-shaped, electron-lucent, vacuole-like intercellular spaces of variable size (approximately 8–13 µm long and 3–9 µm wide). These structures were delimited externally by basal epithelial cells of the outer root sheath and were located adjacent to neurite terminals associated with Merkel cells (Fig 5C).

Within the ringwulst, a densely organized aggregation of fine-caliber nerve fibers that are PGP 9.5- and S100-positive but GFAP- and NF200-negative is present, becoming increasingly dense toward the ring sinus-facing periphery (Figs 3A, 4D, and 4E).

At the attachment site of the ringwulst to the glassy membrane, a further population of medium-caliber nerve fibers immunoreactive for PGP 9.5, NF200, GFAP, and S100 projects toward the glassy membrane (Figs 4E and 4G).

### FSC-innervation at the level of the cavernous sinus (CS)

Within the **cavernous sinus (CS)**, the major branches of the deep vibrissal nerve (DVN) are embedded in connective tissue, ascend through the sinus, and give rise to multiple nerve fibers that project into the sinus’s connective tissue trabeculae as well as into the mesenchymal sheath (MS) (Figs 3A, 4B, 4C, 4F, and 5F). The trabecular fibers are immunoreactive for PGP 9.5, S100, and GFAP; a subset also shows immunoreactivity for NF200. Electron microscopy further reveals lightly myelinated nerve fibers located within the trabeculae (Fig 5H). Within the MS, these DVN-derived fibers ascend along the sheath in a beaded, pearl-like pattern (Fig 4F) and terminate as dense, highly ramifying arborizations of PGP 9.5-, NF200-, S100-, and GFAP-positive nerve fibers along the outer surface of the GM, predominantly oriented perpendicular to the follicle axis (Figs 4C and 5F). Ultrastructurally, the endings consist of branching terminal axons partially ensheathed by Schwann cell processes and surrounded by collagen fibers (Fig 5G). The overall morphology, immunohistochemical phenotype, and orientation of these endings correspond to **reticular nerve endings** described in FSCs of other mammals [17,28].

### Innervation of the hair papilla (HP)

The **hair papilla (HP)** contains a sparse meshwork of fine-caliber PGP 9.5-, S100- and GFAP-positive, NF200-negative nerve fibers, which is confined to the HP and does not extend further proximally along the follicle (Fig 4K).

### General innervation density of canine mystacial FSCs

Characterizing the innervation density of canine (mystacial) FSCs (*n* = 24), PGP 9.5-positive nerve fiber section profiles displayed a total fractional area density of 13.6% ± 3.3% within the internal FSC compartments (follicle-tissue excluding the capsule, the vibrissal hair shaft, rete ridge collar, outer conical body, hair papilla, and sinuses). PGP 9.5-positive nerve tissue area density in mystacial FSCs was not significantly correlated with age or body weight (*r* = 0.2500–0.2938, both *p* > 0.05; S2 Table) and did not differ significantly according to sex, head morphotype, or breed group (all *p* > 0.05; S2 Table).

## Discussion

The results of the present investigation provide reliable evidence that canine FSCs exhibit the typical morphology of sinusoidal follicles across different breeds, ages (including the neonatal stage), and sexes, as well as across different facial regions, as previously demonstrated in several other mammalian species, and contain multiple, densely innervated, specific mechanoreceptors. Using a broad spectrum of analytical methods, including histology, immunohistochemistry, and electron microscopy, we comprehensively characterize the morphology of canine FSCs and the distinct immunoreactivity profiles of specific follicular nerve fiber and mechanoreceptor types across different facial regions, breeds (including poodles and poodle mixes), sexes, and ages. The (immuno)histological analysis of whole FSCs, especially the preparation of histological sections from paraffin-embedded tissue samples, has proven challenging due to the hard, brittle keratin structure of the thick vibrissal hair shaft, which regularly leads to extensive technical artifacts, such as notches and creases in the section, severely limiting their evaluability. In the present study, we developed methods for routinely preparing large-format paraffin sections up to approximately 5 mm in length in extensive test series, enabling the production of high-quality sections suitable for immunohistochemical analysis of FSCs (described in detail in the Supporting Information, S1 Fig). Mechanoreceptors, identified by immunophenotype, localization, and morphology, were consistently observed in technically adequate sections across all examined dogs, including poodles. In addition, the present study reveals distinct histological and ultrastructural features of canine FSC morphology that have not been described in detail previously. These include the presence of vacuole-like spaces associated with Merkel cell-neurite complexes, the pre-capsular (extrafollicular) branching of the DVN, and a densely organized aggregation of fine-caliber unmyelinated nerve fibers within the ringwulst.

Canine mystacial FSCs measured herein were markedly larger than those reported by Ueda in 1941 [23] (length: 2.0 mm), averaging 4.0 mm in length (range: 1.8–5.8 mm) in formalin-fixed, paraffin-embedded (FFPE) sections. This variation likely reflects differences in body size, as body size has been reported to influence FSC size across mammalian species [3], and this relationship was also supported by the present morphometric analyses, which showed significant positive associations between FSC length and width and body weight across all investigated facial regions. The present study included dogs of different breeds, ages, and sexes, whereas the characteristics of the dog examined by Ueda [23] were not specified. Nevertheless, the observed FSC lengths fall within the range reported for other terrestrial mammals, including guinea pigs (*Cavia porcellus*) [47], rats [17,47], and cats [7,17,47]. The mean number of mystacial FSCs identified in dogs was 14 ± 4 per side, which was lower than the mean vibrissa count reported for the red fox, but comparable to counts reported for several primate species [48]. However, the FSC counts obtained in the present study should be interpreted considering that macroscopic identification may be affected by variation in skin pigmentation, coat density and texture, and FSC size among the examined dogs. Furthermore, vibrissal number alone does not account for interspecific differences in vibrissal arrangement, size, stiffness, structure, mobility, or follicular innervation [48]. In contrast to the positive association observed between FSC length and width and body weight, total FSC counts were not significantly associated with body weight. Based on the present data, body size appears to be reflected primarily in FSC dimensions rather than in a general increase in FSC number. The observed considerable interindividual variation in mandibular FSC counts is noteworthy, but its biological significance remains unclear. In the present exploratory analyses, mandibular FSC counts were not significantly associated with body weight or the examined individual or breed-related variables. Thus, this variation does not appear to reflect a simple scaling effect with body size. Further studies, using microscopic FSC enumeration in larger breed-balanced cohorts and additional craniofacial measurements, are required to determine whether mandibular vibrissal number is related to breed-associated craniofacial morphology, selective breeding, individual variation, or specific sensory demands.

Striated muscle fibers attached to the follicle capsule were observed in all four facial regions, from the level of the ring sinus to the lower third of the cavernous sinus. In mystacial FSCs, these fibers represent intrinsic musculature [24,49–52] that enables active, voluntary movement of the vibrissae [27,53,54]. FSCs from non-mystacial facial regions are also associated with striated muscle fibers. However, based on findings in other mammals, these fibers may represent extrinsic facial musculature and may contribute to more generalized movements of the surrounding skin rather than to individual control of the vibrissae [54,55]. Within the canine FSC, a pronounced thickening of the glassy membrane reaching up to approximately 70 µm was observed at the level of the ring sinus in FFPE sections. Additionally, canine FSCs were shown to contain prominent sebaceous glands that completely encircle the follicle, a feature also described in feline FSCs [17]. Another distinct feature of the canine FSC was the pre-capsular bifurcation of the DVN, representing a structural variant of the otherwise unbranched single-DVN condition typical of terrestrial mammals [3,17,50]. This bifurcation creates two branches entering the follicle, a configuration that shows morphological similarity to the dual-DVN arrangement reported in specialized tactile species such as the Florida manatee and the rock hyrax [20,28].

In other mammalian species, distinct mechanoreceptors have been described at specific locations within the follicle-sinus complex. Consistent with these observations, the present study identified Merkel cells, lanceolate endings, reticular endings, and presumptive free nerve endings in the canine FSC, including poodles. **Merkel cells (MCs)** are specialized epithelial cells associated with sensory nerve terminals that represent a well-established class of slowly adapting, low-threshold mechanoreceptors [56,57]. Across mammalian species, such as rodents [3,17], cats [3,17,58], and dogs [25,26], Merkel cells have been consistently identified in two epithelial compartments of the FSC: the rete ridge collar (RRC) and the ring sinus (RS) region. Merkel cells are commonly characterized by their close association with PGP 9.5- and NF200-positive afferent neurites [28,59] and by a distinctive ultrastructural morphology, which includes dense-core granules and finger-like cytoplasmic processes [2,4,18,47,58]. Functionally, Merkel cells primarily respond to static displacement and direction of vibrissal deflection [7,60,61]. Based on their spatial distribution, RRC-associated MCs are thought to respond to large-angle deflections due to their greater distance from the hair shaft, complementing the finer, direction-specific deflection encoding attributed to RS-associated MCs [3,7,28]. Notably, electron-lucent, vacuole-like spaces of various shapes and sizes were observed directly adjacent to the afferent nerve terminals of Merkel cell-neurite complexes at the level of the RS. Comparable structures have only been previously reported in aquatic mammals and semi-aquatic mammals, such as the California sea lion (*Zalophus californianus*) [19], the Australian water rat (*Hydromys chrysogaster*) [47], the European otter (*Lutra lutra*) [18], and the ringed seal (*Phoca hispida*) [18]. In these species, the vacuole-like spaces were interpreted as vesicle-associated structures representing aquatic specializations that enhance Merkel cell sensitivity to external stimuli in hydrodynamic environments [18]. The reported absence of these structures in terrestrial species, such as the polecat (*Mustela putorius)*, was considered to support this interpretation [18]. However, Ramirez et al. [26] described vacuolated nerve terminals adjacent to MCs in canine FSCs using light microscopy. A comparable observation was made in the present study, supporting the interpretation that the vacuolated nerve terminals identified by light microscopy (Fig 4E(i)) correspond to the vacuole-like spaces located directly adjacent to the afferent nerve terminals of Merkel cell-neurite complexes, as demonstrated by transmission electron microscopy (TEM) (Fig 5C). Together, these findings suggest that this Merkel cell-associated morphology is probably a feature of highly specialized vibrissal FSCs rather than an adaptation restricted to aquatic species. Immunohistochemical analysis revealed that S100 labeling was confined to afferent fibers associated with Merkel cell-neurite complexes, without extending into Merkel cells. Earlier canine studies reported cytoplasmic S100 expression in Merkel cells [25], but these findings were obtained only after enzymatic antigen retrieval, which highlights the method’s sensitivity. In contrast, PGP 9.5 reliably labeled Merkel cell-neurite complexes, consistent with studies in cats [17] and rats [16,17], contradicting earlier assumptions that canine MCs lack this marker [25].

**Longitudinal lanceolate endings** were identified in the canine FSCs by their characteristic palisade-like arrangement within the mesenchymal sheath (MS), closely apposed to the glassy membrane (GM) at the level of the lower inner conical body (ICB) and upper ring sinus (RS), as well as by their PGP 9.5-, S100-, and NF200-positive immunoreactivity and their typical ultrastructural morphology of sharp-edged, elongated lanceolate profiles, consistent with descriptions in other mammalian species [2–4,17,28,62–64]. These rapidly adapting mechanoreceptors are thought to encode dynamic aspects of vibrissal displacement, such as changes in speed or direction [3,61]. As in cats, no circumferential lanceolate endings were observed in the canine FSCs at this level, whereas in rats, medium-caliber nerve fibers form circumferential lanceolate endings at the level of the inner conical body (ICB) [3,17]. However, the dense circumferential plexus of PGP 9.5- and GFAP-positive but NF200-negative fibers within the ICB suggests a predominance of thin, unmyelinated nerve fibers, which are commonly interpreted as presumptive **free nerve endings (FNEs)** [2,3,20,28].

Within the ringwulst (RW), a previously unreported aggregation of fine-caliber nerve fibers with a PGP 9.5- and S100-positive but NF200- and GFAP-negative immunohistochemical profile was observed. This marker profile is consistent with thin, unmyelinated afferent nerve fibers, suggesting that they may also terminate as presumptive **free nerve endings (FNEs)**. FNEs have been described in various locations within the FSCs of other mammalian species, including the rete ridge collar, inner conical body, mesenchymal sheath, trabeculae, and hair papilla [2,3,17,20,28,30,62,64–66]. However, a comparably dense accumulation of FNEs within the ringwulst has not been reported previously. In cats, only sparse fine-caliber fibers have been noted in this region [17], in contrast to the pronounced aggregation observed in canine FSCs. This aggregation increased in density toward the peripheral margin of the ringwulst adjacent to the ring sinus. Although the precise function of these fibers remains uncertain, FNEs have been associated with high-threshold mechanoreception [67] and may contribute to nociceptive or thermosensory signaling within the FSC [3].

At the attachment site of the ringwulst (RW) to the glassy membrane (GM), PGP 9.5-, NF200-, S100-, and GFAP-positive medium-caliber fibers project toward the GM. These endings are consistent with **club endings**, as first described by Ebara et al. [17], based on their location and immunohistochemical profile. Ebara et al. [17] characterized club endings as unbranched terminal specializations of medium-caliber fibers associated with terminal Schwann cells and emphasized that these structures had often been mistaken for transected fibers in thin histological sections [3,15,16,30]. Due to their consistent localization within the RW, which projects into the blood-filled ring sinus, it has been suggested that club endings respond to the displacement of the ringwulst relative to the hair shaft of the follicle [17,20,28].

At the level of the cavernous sinus (CS), we observed dense arborizations of PGP 9.5-, NF200-, S100-, and GFAP-immunoreactive nerve fibers that terminate along the outer surface of the glassy membrane (GM). This finding is consistent with **reticular endings** as described in other mammalian species [15–17,28,65,68]. Their perpendicular orientation to the follicle axis is consistent with descriptions in cats [17] and with their proposed role as slowly adapting mechanoreceptors that detect tensile forces acting perpendicular to the follicle axis [17,20,28]. Previous studies [17] have suggested that these highly ramifying endings may correspond to structures previously interpreted as Ruffini-like endings in earlier ultrastructural studies [4,69]. The present ultrastructural findings support this interpretation by demonstrating terminal axons with complex branching patterns as they approach the glassy membrane, partially ensheathed by Schwann cell cytoplasmic lamellae and externally surrounded by collagen fibers.

In the present study, no encapsulated or spiny endings were observed in the canine FSC at the level of the cavernous sinus (CS), in contrast to previous reports describing encapsulated endings in cats [2,17] and spiny endings in cats [17], rats [17,68], and rock hyraxes [28]. The overall pattern of canine FSC innervation observed in the present study, however, largely corresponds to that described in other mammalian species with sensory innervation distributed across the principal structural compartments of the follicle [3]. However, interspecies differences in the presence, morphology, and distribution of mechanoreceptor endings within specific compartments, such as the cavernous sinus (CS), are well documented. For instance, encapsulated endings have also not been reported in the mystacial FSC of the rat [17], trabecular endings have only been reported in the facial vibrissae of Florida manatees [20], and a relative paucity of innervation within the cavernous sinus has been described in the Syrian hamster (*Mesocricetus auratus*) and the Mongolian gerbil (*Meriones unguiculatus*) [3]. The functional significance of these differences remains unclear but may reflect species-specific adaptations in vibrissal mechanics and behavioral differences in vibrissal use [3]. Further comparative and functional studies are needed to clarify the physiological relevance of these interspecies differences. A fine meshwork of PGP 9.5-, S100-, and GFAP-positive but NF200-negative nerve fibers within the hair papilla (HP) is consistent with the presence of fibers that terminate as presumptive free nerve endings based on their morphology and immunohistochemical profile [28]. The innervation of the HP is confined to the bulb region, which matches descriptions in rats [17] but differs from those in cats [17] and manatees [20], in which hair papilla innervation extends more apically.

Neural tissue accounted for 13.6% (vol.) of the total volume of the internal FSC compartments analyzed (mesenchymal sheath, glassy membrane, trabeculae, ringwulst, and inner conical body). To our knowledge, neural tissue volume density has not previously been quantified in vibrissal FSCs, precluding direct interspecies comparisons based on this parameter. Previous studies across species have nevertheless characterized the FSC as a densely innervated sensory organ, primarily based on axon counts in defined sectional areas [3,18,47,63,70]. Reported neuronal fractions in different innervated tissues vary substantially depending on the methodological approach, ranging from approximately 6.2% in the human fetal ileum, as determined by immunostaining for βIII-tubulin (TUJ1), a neuronal marker [71], up to 40% in the human dental pulp, as determined by three-dimensional volumetric analyses [72]. In the present study, exploratory statistical analyses did not reveal significant associations between PGP 9.5-positive nerve tissue area density in mystacial FSCs and age or body weight. Likewise, no significant differences were detected according to sex, head morphotype, or breed group. Although these analyses should be interpreted cautiously due to the exploratory character and the limited sample size within some subgroups, they provide no indication that the dense innervation of canine mystacial FSCs is restricted to specific breed morphotypes, sexes, age groups, or body sizes. Taken together, the quantitative assessment of nerve fiber area fractions in canine mystacial FSCs supports the concept of a highly innervated, and thus presumably functionally relevant, peripheral sensory structure.

Overall, the present study indicates that canine FSCs exhibit a broad spectrum of mechanoreceptor specializations. While many features are shared with other species, differences in the distribution and morphology of sensory endings indicate elements of species-specific specialization. Further research is needed to understand how these anatomical characteristics translate into physiological response properties and vibrissal-mediated behavior. However, no evidence of structural or sensory reduction was observed in the canine FSC in any of the examined breeds. These findings are relevant to the ongoing animal welfare discussion concerning the cosmetic trimming of canine vibrissae. Within this debate, the structural development and functional relevance of vibrissae in domestic dogs, particularly in poodles and poodle-related breeds, have been questioned [13,14]. However, the behavioral relevance of canine vibrissae has already been demonstrated, including their involvement in exploration, feeding, and protective responses [27]. The present findings provide a morphological basis for this functional relevance by showing that canine FSCs are densely innervated and contain specialized mechanoreceptor structures irrespective of breed, sex, and facial region. Since the vibrissal shaft transmits external mechanical stimuli to the densely innervated follicular sensory apparatus [7], shortening or removing it may alter normal receptor stimulation, even when the follicle itself remains intact. Similar welfare considerations have already influenced the handling of vibrissae in horses, where vibrissal trimming is increasingly restricted or prohibited [73]. Consistent with previous studies [27], our results reinforce that canine vibrissae represent functional sensory organs and should not be removed for cosmetic reasons in order to preserve the dog’s sensory integrity.

## Supporting information

S1 Table Characteristics of the examined dogs. Breed, body weight, age, sex, pathological diagnosis, and vibrissal hair shaft morphology are listed for each dog. The number of identified follicle-sinus complexes (FSCs) is given for each examined facial region. Numeric entries indicate that the respective region was available and suitable for complete counting. **NA**, not available; the respective facial region was not available and therefore could not be evaluated. **IC**, incomplete count; individual FSCs from the respective facial region were examined, whereas the total FSC number in that region could not be reliably assessed. Inclusion in the quantitative analysis of innervation density is indicated. Breed-group and head-morphotype group assignments are provided as descriptive categories for exploratory analyses and were not based on official breed-group classifications or individual cephalic index measurements. **BC**, brachycephalic/short-headed; **MC**, mesaticephalic/intermediate; **DC**, dolichocephalic/long-headed.
(PDF)

S2 Table Exploratory statistical analyses of quantitative follicle-sinus complex parameters. For each analysis, the outcome parameter, tested variable, statistical test, test statistic, sample size, and *p* value are reported. Group summaries are additionally provided where applicable. Sample sizes varied according to data availability. **FSC,** follicle-sinus complex; **CI,** confidence interval; **df,** degrees of freedom; **PGP 9.5,** protein gene product 9.5.
(PDF)

S1 Fig Overview of vibrissal follicle-sinus complex (FSC) processing to facilitate paraffin sectioning. (**A)** Fixation in 4% neutral-buffered formalin (>12 h). **(B-C)** *Depilation pathway:* FSCs are treated with commercially available depilatory cream for 90 min (B), followed by TBS wash and manual extraction of the hair shaft using fine tweezers. **(C)** FSC after hair shaft removal. **(B′)** *Tissue softening pathway:* Incubation in a Titriplex-based solution for 48 h to soften tissue prior to embedding. **(D)** FSCs are embedded and stabilized in polymerized 2% agar. **(E)** Routine paraffin embedding of agar-embedded vibrissal FSCs with longitudinal orientation.
(JPG)

S2 Fig Localization and hair shaft morphology of vibrissae in dogs of different breeds. Bars = 2 cm. **(A)** Border Collie, 7 years, female, No. 13. **(B)** Retriever cross-breed, 11 years, female, No. 11. **(C)** Briard cross-breed, 4 years, male, No. 18. **(D)** German Spitz, 4 years, female, No. 24. **(E)** Poodle cross-breed, 8 years, male, No. 7. **(F)** French Bulldog, 1 year, female, No. 22. Vibrissae were consistently present at four facial locations: mystacial (my), supraorbital (su), mandibular (ma), and buccal (bu). In the dog shown in (**C**), the vibrissal hair shafts were markedly curly.
(JPG)

S3 Fig Histomorphology of canine follicle-sinus complexes (FSCs) from all facial vibrissal regions in different breeds. **(A)** Mystacial FSC, Toy Poodle, No. 2. **(B)** Supraorbital FSC, Miniature Poodle, No. 3. **(C)** Buccal FSC, Havanese, No. 25. **(D)** Mandibular FSC, Rough Collie, No. 14. Cap = capsule; CS = cavernous sinus; DVN = deep vibrissal nerve; GM = glassy membrane; HP = hair papilla; ICB = inner conical body; IRS = inner root sheath; M = striated musculature; MS = mesenchymal sheath; OCB = outer conical body; ORS = outer root sheath; RRC = rete ridge collar; RS = ring sinus; RW = ringwulst; SG = sebaceous gland. HE-stained paraffin sections illustrate the general sinus-type organization. Bars = 100 µm.
(JPG)

S4 Fig Course of the deep vibrissal nerve (DVN). **(A)** Longitudinal section of a mystacial FSC (German Shepherd, No. 15), immunolabeled with PGP 9.5 (pan-neuronal marker). **(B**) Cross-section at the level of DVN entry in a supraorbital FSC (Poodle cross-breed, No. 7). The DVN bifurcates into two branches (open arrows in B). Both penetrate the follicular capsule (solid arrows) and give rise to branches coursing through the cavernous sinus toward the mesenchymal sheath (arrowheads). (A) DAB chromogen (brown); hematoxylin counterstain. (B) Toluidine blue and Safranin O-stained semithin section of epoxy resin-embedded tissue. Bars = 100 µm.
(JPG)
