# Supplementary material for: Histomorphological and ultrastructural analysis of vibrissal follicle-sinus complexes in the dog (Canis lupus familiaris)
Source: PLoS One. 2026 Aug 19;21(8):e0355554. doi: 10.1371/journal.pone.0355554 (PMC13489436; doi:10.1371/journal.pone.0355554)
Supplement: Supplementary file 1 [file pone.0355554.s001.pdf]

No.		Breed	Age (y=years, m=months, w=weeks, d=days)	Sex (m=male, f=female)	Body weight (kg)	Pathological Diagnosis	Number of Identified Follicle-Sinus Complexes (FSCs), NA=not available; IC=incomplete count				Vibrissal Hair Shaft Morphology (c=curly, s=straight)	Included in Quantitative Analysis of Innervation Density	Descriptive group assignment	
							mystacial	supra-orbital	buccal	mandibular			Breed group	Head morphotype group
1	Poodle and poodle-related breeds	Poodle	14 d	not determined	NA	not determined	NA	NA	4	NA	c	No		DC
2		Toy Poodle	10 w	not determined	NA	not determined	31	10	8	NA	c	No		DC
3		Miniature Poodle	3 m	m	2.4	Interstitial pneumonia	15	5	10	13	c	Yes		DC
4		Maltipoo	2 m	m	1.0	Chronic enterocolitis	33	9	7	16	s	No	Poodle/Poodle-related	MC
5		Australian Labradoodle	9 d	not determined	NA	not determined	34	12	5	NA	s	No		MC
6		Australian Labradoodle	20 d	not determined	NA	not determined	48	11	10	NA	s	No		MC
7		Poodle cross-breed	8 y	m	NA	Multicentric lymphoma	18	9	10	19	s	Yes		MC
8	Retrievers	Golden Retriever	4 w 3 d	m	3.7	Necrotizing enterocolitis	32	7	5	4	s	Yes		MC
9		Labrador Retriever	11 d	f	0.3	not determined	31	12	4	10	s	Yes	Hunting- and working-related breeds	MC
10		Labrador Retriever	5 y	m	35.0	Hemorrhagic gastroenteropathy	29	9	4	16	s	Yes		MC
11		Retriever cross-breed	11 y	f	21.1	Metastatic hemangiosarcoma	28	10	7	16	s	Yes		MC
12	Herding Dogs	Dutch Shepherd	5 y	f	27.5	Multisystemic necrotizing vasculitis	34	IC	NA	NA	s	No		MC
13		Border Collie	7 y	f	16.4	Nodular hepatic fibrosis	21	13	5	12	s	Yes		MC
14		Rough Collie	4 y	f	17.7	Hemorrhagic-necrotizing enteritis	35	12	9	28	s	Yes		DC
15		German Shepherd	8 y	m	47.0	Perianal apocrine adenocarcinoma	42	12	4	20	s	Yes	Herding, sled-type and large breeds	MC
16		Shetland Sheepdog	8 w	f	1.0	Parasitic enteritis (coccidiosis)	22	8	4	9	s	No		DC
17		Berger Blanc Suisse	13 y	f	31.4	not determined	32	10	4	11	s	Yes		MC
18		Briard cross-breed	4 y	m	17.8	Interstitial pancreatic hemorrhage	35	9	11	15	c	Yes		MC
19	Brachycephalic breeds	Boxer	0 d	f	0.5	Intrauterine death (undetermined etiology)	17	5	NA	NA	s	No	Hunting- and working-related breeds	BC
20		Boxer	0 d	f	0.3	Bacterial sepsis	15	IC	NA	NA	s	No		BC
21		French Bulldog	9 y	m	15.0	Hemorrhagic myelomalacia	24	7	6	18	s	No		BC
22		French Bulldog	1 y 5 m	f	11.1	not determined	25	9	9	11	s	Yes		BC
23	Companion and small breeds	German Spitz	7 d	f	0.2	Hemorrhagic necrotizing encephalitis	13	NA	NA	NA	s	No	Companion and small breeds	MC
24		German Spitz	4 y	f	11.4	not determined	23	10	10	16	s	No		MC
25		Havanese	13 y	m	5.2	Necrotizing lymphoplasmacytic hepatitis	31	8	8	14	c	Yes		MC
26		Phalene	3 w	m	0.2	Renal dysplasia	17	6	5	16	s	Yes		MC
27	Terriers	Jack Russell Terrier	16 y	f	2.6	Hypertrophic cardiomyopathy	23	10	5	NA	s	No		MC
28		Fox Terrier	10 y	f	9.5	Chronic cardiomyopathy	20	6	7	IC	s	No		DC
29	Hunting and sporting breeds	Irish Red Setter	4 d	m	0.3	Interstitial pneumonia	IC	NA	NA	NA	s	No		DC
30		Beagle cross-breed	8 y	f	29.8	Membranous glomerulonephritis	17	8	4	5	s	Yes		MC
31		Magyar Vizsla	10 y 6 m	m	24.7	Multicentric hemangiosarcoma	31	9	9	14	s	Yes		MC
32		Dachshund	11 y	f	8.8	Suppurative-necrotizing myocarditis	27	8	8	18	s	No		DC
33		Wirehaired Dachshund	14 y	f	11.0	Subcutaneous hemorrhagic panniculitis	IC	9	7	IC	s	No	Hunting- and working-related breeds	DC
34		German Quail Dog	6 m	m	18.7	Thymic hemorrhage with hemothorax	27	7	6	32	s	Yes		MC
35		German Shorthaired Pointer	1 y 2 m	f	14.0	Lymphoplasmacytic enterocolitis	34	11	5	28	s	No		MC
36		English Pointer cross-breed	13 y	m	19.6	Cerebral atrophy	27	10	10	20	s	Yes		DC
37		Rhodesian Ridgeback	5 w	m	3.6	Mesenteric venous thrombosis	32	10	10	21	s	Yes		DC
38		Ca Rater Mallorquí	12 y	m	6.6	Membranoproliferative and interstitial nephritis	28	10	10	14	s	No		MC
39		Danish-Swedish Farmdog	2 y 6 m	m	11.8	Systemic hemorrhagic syndrome	31	5	10	8	s	Yes		MC
40	Large breeds	Saint Bernard	8 y	m	76.0	Congestive cardiomyopathy	22	5	8	4	s	Yes		MC
41		Siberian Husky	7 y	m	25.2	Multicentric hemangiosarcoma	30	11	5	14	s	Yes		MC
42		Great Dane	4 y	f	67.0	Thoracolumbar myelopathy	42	7	9	33	s	Yes	Herding, sled-type and large breeds	DC
43		Bernese Mountain Dog	9 y	f	35.2	Traumatic hemothorax with pulmonary hemorrhage	26	9	6	15	s	Yes		MC
44		Mixed-breed (large-sized)	3 y	m	34.7	Fibrinosuppurative necrotizing bronchopneumonia	33	7	8	IC	s	No		MC
