# Supplementary material for: Histomorphological and ultrastructural analysis of vibrissal follicle-sinus complexes in the dog (Canis lupus familiaris)
Source: PLoS One. 2026 Aug 19;21(8):e0355554. doi: 10.1371/journal.pone.0355554 (PMC13489436; doi:10.1371/journal.pone.0355554)
Supplement: Supplementary file 2 [file pone.0355554.s002.pdf]

Outcome parameter	Tested variable	Statistical test	Test statistic / group summary	*n*	*p* value
Mandibular FSC count	Age (years)	Spearman rank correlation	*r* = 0.0493, 95% CI −0.3203 to 0.4059	31	0.7922
Mandibular FSC count	Body weight (kg)	Spearman rank correlation	*r* = 0.1365, 95% CI −0.2459 to 0.4821	30	0.4721
Mandibular FSC count	Mandibular FSC length (mm)	Spearman rank correlation	*r* = 0.1008, 95% CI −0.2731 to 0.4482	31	0.5895
Mandibular FSC count	Sex	Mann-Whitney *U* test	Female median = 15.0, *n* = 13; male median = 15.5, *n* = 18; *U* = 111	31	0.8200
Mandibular FSC count	Head morphotype	Kruskal-Wallis test	Kruskal–Wallis statistic = 3.170; df = 2	31	0.2049
Mandibular FSC count	Breed group	Kruskal-Wallis test	Kruskal–Wallis statistic = 0.1986; df = 3	31	0.9778
Total FSC count	Age (years)	Spearman rank correlation	*r* = 0.1215, 95% CI −0.2536 to 0.4648	31	0.5149
Total FSC count	Body weight (kg)	Spearman rank correlation	*r* = 0.1864, 95% CI −0.1972 to 0.5204	30	0.3241
Total FSC count	Sex	Mann-Whitney *U* test	Female median = 57.0, *n* = 13; male median = 60.5, *n* = 18; *U* = 111	31	0.8205
Total FSC count	Head morphotype	Kruskal-Wallis test	Kruskal–Wallis statistic = 2.361; df = 2	31	0.3072
Total FSC count	Breed group	Kruskal-Wallis test	Kruskal–Wallis statistic = 2.094; df = 3	31	0.5530
Mystacial FSC length (mm)	Body weight (kg)	Spearman rank correlation	*r* = 0.7871, 95% CI 0.6215 to 0.8854	39	< 0.001
Mystacial FSC width (mm)	Body weight (kg)	Spearman rank correlation	*r* = 0.8754, 95% CI 0.7695 to 0.9344	39	< 0.001
Supraorbital FSC length (mm)	Body weight (kg)	Spearman rank correlation	*r* = 0.7494, 95% CI 0.5550 to 0.8662	37	< 0.001
Supraorbital FSC width (mm)	Body weight (kg)	Spearman rank correlation	*r* = 0.8415, 95% CI 0.7065 to 0.9174	37	< 0.001
Buccal FSC length (mm)	Body weight (kg)	Spearman rank correlation	*r* = 0.7712, 95% CI 0.5790 to 0.8822	34	< 0.001
Buccal FSC width (mm)	Body weight (kg)	Spearman rank correlation	*r* = 0.7127, 95% CI 0.4855 to 0.8497	34	< 0.001
Mandibular FSC length (mm)	Body weight (kg)	Spearman rank correlation	*r* = 0.7263, 95% CI 0.5023 to 0.8589	33	< 0.001
Mandibular FSC width (mm)	Body weight (kg)	Spearman rank correlation	*r* = 0.7089, 95% CI 0.4750 to 0.8492	33	< 0.001
PGP 9.5-positive nerve tissue area density in mystacial FSCs (%)	Age (years)	Spearman rank correlation	*r* = 0.2938, 95% CI −0.1367 to 0.6310	24	0.1634
PGP 9.5-positive nerve tissue area density in mystacial FSCs (%)	Body weight (kg)	Spearman rank correlation	*r* = 0.2500, 95% CI −0.1933 to 0.6086	23	0.2499
PGP 9.5-positive nerve tissue area density in mystacial FSCs (%)	Sex	Mann-Whitney *U* test	Female median = 15.59%, *n* = 9; male median = 12.35%, *n* = 15; *U* = 42	24	0.1383
PGP 9.5-positive nerve tissue area density in mystacial FSCs (%)	Head morphotype	Kruskal-Wallis test	Kruskal–Wallis statistic = 0.8604; df = 2	24	0.6504
PGP 9.5-positive nerve tissue area density in mystacial FSCs (%)	Breed group	Kruskal-Wallis test	Kruskal–Wallis statistic = 1.542; df = 3	24	0.6726
